# Discordance of Circulating Non-HDL Cholesterol with LDL Cholesterol Concerning Long-Term Prognosis in Statin-Treated Individuals with Acute Coronary Syndrome and Previous Coronary Artery Bypass Grafting Undergoing Percutaneous Coronary Intervention

**DOI:** 10.31083/j.rcm2409263

**Published:** 2023-09-21

**Authors:** Chuang Li, Kuizheng He, Yixing Yang, Kuibao Li, Mulei Chen, Lefeng Wang, Yuanfeng Gao, Xiaorong Xu

**Affiliations:** ^1^Heart Center and Beijing Key Laboratory of Hypertension, Beijing Chaoyang Hospital, Capital Medical University, 100020 Beijing, China

**Keywords:** discordance, non-HDL-C, prior coronary artery bypass grafting, statin therapy, major adverse cardiovascular events

## Abstract

**Background:**

Some individuals who maintain desirable low-density lipoprotein cholesterol (LDL-C) levels still experience the progression of atherosclerosis, which may eventually lead to cardiovascular events. Non-high-density lipoprotein cholesterol (non-HDL-C) levels are quantified to assess residual risk in statin-treated patients with coronary heart disease. The study aimed to estimate the predictive performance of discordance between non-HDL-C and LDL-C on clinical prognosis in statin-treated patients with previous coronary artery bypass grafting (CABG).

**Methods:**

468 statin-treated patients with previous CABG undergoing percutaneous coronary intervention (PCI) as a secondary coronary treatment due to acute coronary syndrome (ACS) were retrospectively enrolled in this study. The definition of major adverse cardiovascular events (MACEs) was a composite endpoint of cardiovascular death, recurring myocardial infarction, and a need for repeat revascularization. Cox proportional hazards modeling, restricted cubic splines regression, and discordance analysis were conducted to the association between all lipid parameters and the occurrence of MACEs. Discordant values were defined as LDL-C concentrations ≤1.8 mmol/L accompanied by non-HDL-C >2.6 mmol/L.

**Results:**

MACEs occurred in 95 patients over a median follow-up period of 744.5 days. Cox models demonstrated that increased concentrations of non-HDL-C and LDL-C levels were independent risk indicators of MACEs (*p* < 0.001). The restricted cubic spline analysis revealed a linear relationship between non-HDL-C concentrations and MACEs (p-nonlinear: 0.26), whereas a nonlinear relationship was observed between LDL-C concentrations and MACEs (*p* < 0.01). In the subgroup analysis, the spline curves revealed that the odds of the individuals with desirable LDL-C levels suffering MACEs emerged when non-HDL-C levels were above 2.07 mmol/L. Individuals who exhibited discordance involving high non-HDL-C/low LDL-C levels had an elevated risk of experiencing MACEs compared to those with concordantly low LDL-C and low non-HDL-C levels [hazard ratios (HRs) = 2.44, 95% confidence interval (CI) = 1.14–5.22, *p* = 0.02].

**Conclusions:**

Non-HDL-C levels could predict the residual risk of MACEs in ACS patients with previous CABG and statin therapy that underwent percutaneous coronary intervention. A discordance between non-HDL-C and LDL-C in individuals with desirable LDL-C levels could be useful in identifying those with a residual risk of cardiovascular complications.

## 1. Introduction

Generally, low-density lipoprotein cholesterol (LDL-C) levels above a certain threshold are a well-known risk indicator for contributing to atherosclerotic cardiovascular disease (ASCVD). Lowering such levels is a paramount therapeutic goal of the guidelines drafted for managing hypercholesterolemia [1, 2]. However, in high triglyceride levels and metabolic diseases such as obesity, metabolic syndrome, gout, and diabetes mellitus, some individuals who maintain desirable LDL-C values still encounter the exacerbation of atherosclerosis, which may eventually lead to cardiovascular events [3, 4, 5]. Additionally, non-high-density lipoprotein cholesterol (non-HDL-C) can contribute to the atherogenic risk caused by various atherogenic risk components of remnant lipoprotein particles and is an acceptable surrogate marker for apolipoprotein B [3, 6, 7]. An increasing body of evidence, supported by earlier studies, has demonstrated that measuring non-HDL-C levels is superior to quantifying LDL-C in identifying statin-treated individuals with a higher residual risk of ASCVD who may require more intensive therapy; such measurements are already recommended by treatment guidelines [4, 8, 9, 10]. Many studies have discovered that a certain proportion of individuals with optimal LDL-C levels exhibit unexpectedly high non-HDL-C levels, coupled with high triglyceride levels, or metabolic syndrome and diabetes [5, 11]. Previous studies have postulated that the phenomenon of high non-HDL-C with low LDL-C is termed as the discordance and may reflect a greater residual ASCVD risk, regardless of whether an individual is undergoing lipid-lowering therapy [8, 10].

Patients with previous coronary artery bypass grafting (CABG) surgery experience an accelerated progression of atherosclerosis, which can further increase the incidence of recurrent cardiovascular (CV) events [12, 13, 14]. A previous study has discovered a better predictive value of non-HDL-C in terms of CV risk than LDL-C levels in this population. However, most of the participants in that study were not undergoing routine statin treatment [15]. Therefore, few studies have investigated whether a discordance of non-HDL-C levels and LDL-C levels correlated with elevated CV risk in statin-treated individuals with previous CABG compared with the risk in those with concordant levels of the two factors. Given these contemplations, the first objective of this study is to examine the association of serum concentrations of non-HDL-C with CV events among post-CABG individuals who received secondary percutaneous coronary intervention (PCI) treatment. The second objective was to determine whether a discordance between non-HDL-C and LDL-C levels affects prognosis.

## 2. Materials and Methods

### 2.1 Study Participants and Inclusion Criteria

We retrospectively recruited 480 consecutive participants with previous CABG from 14,288 patients who underwent coronary interventions as secondary revascularization due to acute coronary syndrome (ACS) at Beijing Chaoyang Hospital between January 2015 and December 2020. The inclusion criteria included: (i) diagnosis of ACS, including the presence of symptoms related to coronary ischemia and ST-segment elevation or depression on the electrocardiogram (ECG), with or without elevated levels of cardiac troponins; and (ii) an initial dose of a cholesterol-lowering drug for 6–8 weeks before admission prescribed for all eligible participants based on their risk-stratification and lipid-lowering efficiency of the drugs. The patients’ risk-stratification and target LDL-C levels were based on the management of dyslipidemia guidelines recommended by the 2019 European Society of Cardiology/European Atherosclerosis Society (ESC/EAS) [1]. The excluded standard includes a shortage of therapeutic statin data, an expected remaining lifespan of 6 months or less, and end-stage of liver cirrhosis. A total of 468 statin-treated participants with a history of CABG, who underwent PCI, were finally included in this retrospective study, while 7 cases with incomplete statin course and 5 cases with missing follow-up were excluded (see **Supplementary Fig. 1**). This study complied with the principles of the Declaration of Helsinki and the Beijing Chaoyang Hospital institution’s ethical policies. The informed consent was dispensable because of the retrospective feature of the study.

### 2.2 Collection of Demographic Data and Calculation of Lipid Parameters 

Demographic data were gathered from Beijing Chaoyang Hospital’s electronic database and included age, gender, atherosclerotic risk factors, major diagnosis, laboratory variables, vital signs at discharge, and postoperative medication. The fasting serum levels of total cholesterol (TC), HDL-C, triglyceride (TG), and apolipoprotein A were measured within 24 hours of admission and used to help calibrate the doses of lipid-lowering drugs to achieve LDL-C goals for different patients. LDL-C was calculated by the classical Friedewald method [16]. The calculation of non-HDL-C at admission was conducted as total cholesterol minus HDL-C. Meanwhile, we also collected detailed information regarding the history of PCI before or after CABG. The clinical risk in the setting of ACS and previous CABG was estimated by two independent clinicians based on the Global Registry of Acute Coronary Events (GRACE) risk score [17].

The target of lipid-lowing treatment for patients with multivessel disease was LDL-C ≤1.8 mmol/L and non-HDL-C ≤2.6 mmol/L, respectively, according to the recommendations of the guidelines mentioned above. All enrolled patients after enrolment in this study were stratified into two mutually exclusive concordance/discordance groups: low/low (LDL-C level ≤1.8 mmol/L and non-HDL-C level ≤2.6 mmol/L), low/high (LDL-C level ≤1.8 mmol/L and non-HDL-C level >2.6 mmol/L), high/low (LDL-C level >1.8 mmol/L and non-HDL-C level ≤2.6 mmol/L), and high/high (LDL-C level >1.8 mmol/L and non-HDL-C level >2.6 mmol/L).

### 2.3 Intervention Procedure and Coronary Complication 

Angiography and angioplasty were implemented by a senior interventionist who had independently completed at least 300 interventions per year. The interventionist implemented an intervention strategy that includes drug-coated balloon (DCB) angioplasty, stent implantation, or plain old balloon angioplasty (POBA) according to the type of lesion. Coronary angiography and intervention procedure were retrospectively reviewed by two cardiologists (Dr. Li and Dr. He); the factors included the culprit vessel, type of angioplasty, and serious coronary complications. Serious coronary complications include acute coronary occlusion because of in-stent thrombus or coronary dissection and coronary penetration.

### 2.4 Outcomes Ascertainment 

The term major adverse cardiovascular events (MACEs) was a composite endpoint of cardiac mortality, recurrent non-fatal myocardial infarction (MI), and the need for repeated revascularization (defined as that driven by symptoms of clinical ischemia). However, the planned staged PCI was not taken into consideration for revascularization. The documentation of individuals’ clinical adverse events was conducted via telephone conversations, outpatient visits, or inpatient records.

### 2.5 Statistical Analysis

Continuous variables were summarized as median (interquartile range [IQR]) and categorical variables as frequency and percentage. The Student’s *t*-test, analysis of variance (ANOVA), and Chi-Squared test were performed for continuous and categorical variables, as appropriate, to compare baseline characteristics among groups. Kaplan-Meier method with log-rank test was used to estimate survival discrepancies among different lipid groups. The Cox regression analysis was performed to determine the predictive values of the serum lipid parameters as a continuous scale for MACEs after modifying for baseline age, sex, body mass index (BMI), conventional risk variables, vital signs at discharge, vein grafting PCI, and lipid-lowering therapy. Hazard ratios (HRs) were applied for standardized increments of 1 standard deviation (SD) of the continuous variables to estimate the independent association of different serum lipid indices with clinical CV events. Restricted cubic splines (RCS) with three knots at the 5th, 50th, and 95th percentiles were performed to explore and visualize the relation of LDL-C or non-HDL-C levels with different CV outcomes in the setting of previous CABG and statin therapy based on the above Cox proportional hazards models [18].

Meanwhile, cubic splines were described between non-HDL-C and outcomes at low/high LDL levels to explore the cut-off values. The variance inflation factor (VIF) was used to identify multicollinearity, and a VIF <10 indicated the possibility of low intercorrelations among independent parameters in the multivariable regression model. The Cox model was applied to estimate whether the discordance was related to the occurrence of MACE in the discordance analyses. All statistical analyses were done using R version 4.1.0 software (R Foundation for Statistical Computing, Vienna, Austria), and a two-tailed *p*-value of ≤0.05 was considered statistically significant.

## 3. Results

### 3.1 Clinical and Procedural Baseline Characteristics 

All participants were categorized into the following two groups based on the occurrence of CV complications: the MACE group and the non-MACE group. The patients’ baseline clinical and procedural characteristics are shown in Table 1. Participants in the MACE group had a more elevated frequency of saphenous grafting interventions (*p* = 0.06) and stent implantation (*p* < 0.01) compared with the non-MACE group regarding procedural characteristics. Meanwhile, a similar incidence of coronary complications occurred among these two groups.

**Table 1.** **Baseline characteristic**.

Factor			Total	MACE	Non-MACE	*p*-value
N (%)			468	95 (20.2%)	373 (70.5%)	
Age (year)			68 (62, 75)	68 (62, 76)	68 (62, 75)	0.93
Male, n (%)			378 (80.8%)	74 (77.9%)	304 (86.3%)	0.51
BMI (kg/$m^{2}$)			26.3 (24.2, 28.3)	26.6 (24.7, 28.3)	26.2 (24.2, 28.4)	0.51
Diagnosis, n (%)						<0.01
	STEMI		56 (12.0%)	23 (24.2%)	33 (8.9%)	
	NSTEMI		83 (17.7%)	12 (12.6%)	71 (19.0%)	
	Unstable angina		329 (70.3%)	60 (63.2%)	269 (72.1%)	
LVEF, %			62 (55, 68)	61 (55, 67)	62 (55, 68)	0.39
Discharge heart rate (beats/min)			69 (61, 76)	67 (61, 75)	70 (61, 76)	0.24
Discharge systolic blood pressure (mmHg)			134 (19.1)	134 (19.5)	134 (18.9)	0.89
Discharge diastolic blood pressure (mmHg)			74 (67, 80)	74 (67, 80)	73 (67, 80)	0.93
Previous MI, n (%)			145 (31.0%)	33 (34.7%)	112 (30.0%)	0.45
No history of PCI, n (%)			321 (68.6%)	61 (64.2%)	260 (69.7%)	0.41
History of PCI before CABG, n (%)			38 (8.1%)	7 (7.4%)	31 (8.3%)	
History of PCI after CABG, n (%)			109 (23.3%)	27 (28.4%)	82 (22.0%)	
History of stroke, n (%)			69 (14.7%)	13 (13.7%)	56 (15.0%)	0.18
Diabetes mellitus, n (%)			213 (45.5%)	43 (45.3%)	170 (45.6%)	1.0
Hypertension, n (%)			349 (74.6%)	71 (74.7%)	278 (74.5%)	1.0
Smoker, n (%)			278 (59.4%)	62 (65.3%)	216 (57.9%)	0.24
Procedural characteristic						
	Culprit artery, n (%)					0.06
		LAD, n (%)	79 (16.9%)	13 (13.7%)	66 (17.7%)	
		LCX, n (%)	104 (22.2%)	20 (21.1%)	84 (22.5%)	
		RCA, n (%)	173 (37.0%)	29 (30.5%)	144 (38.6%)	
		LM, n (%)	32 (6.8%)	7 (7.4%)	25 (6.7%)	
		AO-SVG-LAD, n (%)	12 (2.6%)	2 (2.1%)	10 (2.7%)	
		AO-SVG-LCX, n (%)	34 (7.3%)	13 (13.7%)	21 (5.6%)	
		AO-SVG-RCA, n (%)	34 (7.3%)	11 (11.6%)	23 (6.2%)	
	Type of angioplasty					<0.01
		DES, n (%)	354 (75.6%)	86 (90.5%)	268 (71.8%)	
		DCB, n (%)	105 (22.4%)	9 (9.4%)	96 (25.7%)	
		POBA, n (%)	9 (1.9%)	0 (0.0%)	9 (2.4%)	
Coronary complication						
	Coronary dissection, n (%)		5 (1.1%)	0 (0.0%)	5 (1.3%)	0.56
	Acute in-stent thrombus, n (%)		1 (0.2%)	1 (1.0%)	0 (0.0%)	0.46
	Coronary penetration, n (%)		2 (0.4%)	1 (1.0%)	1 (0.2%)	0.87
Statin treatment						0.92
	Atorvastatin, n (%)		374 (79.9%)	75 (78.9%)	299 (80.2%)	
	Rosuvastatin, n (%)		88 (18.8%)	19 (20.0%)	69 (18.5%)	
	Other statins, n (%)		6 (1.3%)	1 (1.1%)	5 (1.3%)	
Laboratory test						
	Hemoglobin (g/L)		131 (17.3)	132 (16.2)	131.2 (17.6)	0.61
	Total cholesterol (mmol/L)		3.57 (3.09, 4.22)	3.69 (3.09, 4.37)	3.56 (3.11, 4.18)	0.46
	LDL-C (mmol/L)		1.9 (1.58, 2.46)	1.9 (1.50, 2.55)	1.9 (1.59, 2.4)	0.98
	HDL-C (mmol/L)		0.96 (0.80, 1.10)	1.00 (0.80, 1.10)	0.95 (0.80, 1.10)	0.62
	Triglyceride (mmol/L)		1.37 (0.97, 1.93)	1.49 (0.97, 2.08)	1.36 (0.97, 1.89)	0.59
	Non-HDL-C (mmol/L)		2.60 (2.11, 3.20)	2.70 (2.14, 3.34)	2.56 (2.09, 3.16)	0.21
	Lipoprotein (a) (mmol/L)		19.6 (9.0, 37.5)	21.6 (11.1, 44.4)	19.1 (8.8, 35.0)	0.30
	HbA1c (%)		6.65 (6.0, 7.93)	6.50 (5.90, 7.75)	6.70 (6.00, 8.00)	0.48
	Brain natriuretic peptide (pg/mL)		185 (73, 647)	208 (83, 957)	184 (72, 565)	0.13
	Hs-CRP (mg/dL)		2.74 (0.99, 4.77)	2.36 (0.92, 4.83)	2.91 (1.00, 4.77)	0.74
	ESR (mm/h)		6.0 (2.0, 12.2)	6 (2, 13)	6 (2, 12)	0.86
	Creatinine (umol/L)		78.1 (66.9, 90.8)	83.7 (70.7, 97.1)	76.4 (66.4, 89.2)	<0.01
	GRACE risk score		95 (79, 112)	94 (79, 110)	94 (79, 110)	0.38
Medication at discharge						
	Aspirin, n (%)		464 (99.2%)	94 (98.9%)	370 (99.2%)	1.0
	Clopidogrel, n (%)		459 (98.1%)	95 (100.0%)	364 (97.6%)	0.06
	ACEI/ARB, n (%)		178 (38.0%)	33 (34.7%)	145 (38.9%)	<0.01
	β-blocker, n (%)		347 (74.2%)	62 (65.3%)	285 (76.4%)	0.04

Note: N and n, numbers of eligible patients; BMI, body mass index; NSTEMI, non ST-segment elevated myocardial infraction; STEMI, ST-segment elevated myocardial infraction; LVEF, left ventricular ejection fraction; MI, myocardial infarction; PCI, percutaneous coronary intervention; LAD, left anterior descending coronary artery; LCX, left circumflex coronary artery; RCA, right coronary artery; LM, left main coronary artery; AO, ascending aorta; SVG, saphenous vein grafting; DES, drug-eluting stent; DCB, drug-coated balloon; POBA, plain old balloon angioplasty; LDL-C, low-density lipoprotein cholesterol; HDL-C, high-density lipoprotein cholesterol; Hs-CRP, high-sensitivity C-reactive protein; ESR, erythrocyte sedimentation rate; GRACE risk score, global registry of acute coronary events risk score; ACEI, angiotensin converting enzyme inhibitors; ARB, angiotensin receptor blocker; MACE, major adverse cardiovascular event; CABG, coronary artery bypass grafting; HbA1c, hemoglobinA1c.

The median LDL-C and non-HDL-C concentrations were 1.90 mmol/L and 2.60 mmol/L among all patients receiving statins therapy (including 79.9% atorvastatin, 18.8% rosuvastatin, and 1.3% other statins), respectively. Of all patients with previous CABG, 76 (16.2%) presented discordant, and 24 (5.1%) with low LDL-C had increasing levels of non-HDL-C (shown in Table 2). A significantly lower proportion of the patients with discordantly high non-HDL-C and low LDL-C levels were male than female (*p* = 0.02), and they exhibited an increased erythrocyte sedimentation rate (*p* < 0.001) and a higher BMI (*p* = 0.02). The treatment frequencies with beta-blockers and statin therapy were similar, without significant discrepancy in other characteristics among the discordance/concordance groups.

**Table 2.** ** Baseline characteristic of the discordance/concordance groups**.

Factor		Low LDL-C (≤1.8 mmol/L)		High LDL-C (>1.8 mmol/L)		*p*-value
		Low non-HDL-C (≤2.6 mmol/L)	High non-HDL-C (>2.6 mmol/L)	Low non-HDL-C (≤2.6 mmol/L)	High non-HDL-C (>2.6 mmol/L)	
N (%)		183 (43.8%)	24 (5.1%)	52 (11.1%)	209 (44.7%)	
Age (year)		67 (62, 73.5)	69 (65.8, 75.3)	68 (61, 77.3)	69 (62, 76)	0.58
Male, n (%)		159 (86.9%)	16 (66.7%)	44 (84.6%)	159 (76.1%)	0.02
BMI (kg/$m^{2}$)		25.8 (23.7, 27.7)	27.0 (24.8, 28.3)	26.1 (24.9, 27.7)	26.6 (24.6, 28.7)	0.02
Diagnosis, n (%)						0.46
	STEMI	16 (8.7%)	3 (12.5%)	6 (11.5%)	31 (14.8%)	
	NSTEMI	28 (15.3%)	4 (16.7%)	10 (19.2%)	41 (19.6%)	
	Unstable angina	139 (75.9%)	17 (70.8%)	36 (69.2%)	137 (65.6%)	
LVEF, %		62 (55, 68)	62 (55.8, 66)	60.5 (53.7, 68)	62 (55, 68)	0.63
Discharge heart rate (beats/min)		68 (61, 75)	66.5 (62.3, 77)	70 (60.8, 78.0)	68 (62, 78)	0.84
Discharge systolic blood pressure (mmHg)		132 (17.6)	138 (20.8)	137 (20.9)	134 (19.6)	0.32
Discharge diastolic blood pressure (mmHg)		75 (67, 80)	78 (64.8, 80.3)	72 (67.8, 80.0)	72 (67, 80)	0.93
Previous MI, n (%)		54 (29.5%)	4 (16.7%)	23 (44.2%)	64 (30.6%)	0.07
No previous PCI, n (%)		54 (29.5%)	7 (29.2%)	17 (32.6%)	69 (33.0%)	0.54
Previous PCI before CABG, n (%)		14 (7.6%)	3 (12.5%)	7 (13.5%)	14 (6.7%)	
Previous PCI after CABG, n (%)		40 (21.9%)	4 (16.7%)	10 (19.2%)	55 (26.3%)	
History of stroke, n (%)		24 (13.1%)	3 (12.5%)	7 (13.46%)	35 (16.8%)	0.75
Diabetes mellitus, n (%)		85 (46.4%)	11 (45.8%)	22 (42.3%)	95 (45.5%)	0.96
Hypertension, n (%)		131 (71.6%)	19 (79.2)	37 (71.1%)	162 (77.5%)	0.49
Smoker, n (%)		117 (63.9%)	15 (62.5%)	26 (50.0%)	120 (57.4%)	0.27
Statin treatment						0.43
	Atorvastatin, n (%)	156 (85.3%)	19 (79.2%)	40 (76.9%)	159 (76.1%)	
	Rosuvastatin, n (%)	25 (13.6%)	5 (20.8%)	11 (21.2%)	47 (22.5%)	
	Other statins, n (%)	2 (1.0%)	0 (0%)	1 (1.9%)	3 (1.4%)	
Laboratory test						
	Hemoglobin (g/L)	129 (17.5)	127 (19.5)	134 (15.3)	133 (17.0)	0.03
	Total cholesterol (mmol/L)	3.04 (2.66, 3.29)	3.84 (3.56, 4.49)	3.38 (3.21, 3.56)	4.24 (3.87, 4.66)	<0.001
	HDL-C (mmol/L)	0.95 (0.80, 1.11)	0.80 (0.79, 1.06)	0.96 (0.80, 1.20)	1.0 (0.84, 1.10)	0.22
	Triglyceride (mmol/L)	1.04 (0.77, 1.46)	2.86 (1.49, 4.10)	1.20 (0.89, 1.59)	1.64 (1.23, 2.34)	<0.001
	Lipoprotein (a) (mmol/L)	19.5 (8.4, 34.2)	22.0 (10.3, 34.4)	24.2 (10.33, 36.6)	18.8 (9.2, 39.6)	0.63
	HbA1c (%)	6.60 (6.00, 7.90)	7.10 (6.30, 8.20)	6.3 (5.87, 7.12)	6.70 (6.00,8.00)	0.11
	Brain natriuretic peptide (pg/mL)	208 (83, 718)	103 (51, 488)	182 (75, 675)	174 (73, 616)	0.53
	Creatinine (umol/L)	79.0 (68.2, 90.3)	82.5 (66.8, 104.7)	77.1 (66.9, 85.6)	76.8 (65.9, 91.5)	0.54
	Hs-CRP (mg/dL)	2.15 (0.77, 4.77)	2.99 (0.79, 4.77)	2.58 (0.80, 4.77)	3.26 (1.31, 4.77)	0.10
	ESR (mm/h)	4 (2, 10)	10 (3.5, 15)	5 (2, 12.3)	9 (3, 15)	<0.001
	GRACE risk score	92 (79, 103)	88 (78.5, 111.5)	95 (82.5, 113.5)	97 (79, 114)	0.28
Discharge medication						
	Aspirin, n (%)	182 (99.5%)	24 (100%)	51 (98.1%)	207 (99.04)	0.76
	Clopidogrel, n (%)	182 (99.5%)	24 (100%)	50 (96.2%)	203 (97.0%)	0.22
	β-blocker, n (%)	136 (74.3%)	16 (66.7%)	38 (73.1%)	157 (75.1%)	0.84
	ACEI/ARB, n (%)	62 (33.9%)	13 (54.2%)	22 (42.3%)	81 (38.7%)	0.21

Abbreviation: N and n, numbers of patients; BMI, body mass index; STEMI, ST-segment elevated myocardial infraction; NSTEM, non ST-segment elevated myocardial infraction; LVEF, left ventricular ejection fraction; MI, myocardial infarction; PCI, percutaneous coronary intervention; LDL-C, low-density lipoprotein cholesterol; HDL-C, high-density lipoprotein cholesterol; Hs-CRP, high-sensitivity C-reactive protein; ESR, erythrocyte sedimentation rate; GRACE risk score, global registry of acute coronary events risk score; ACEI, angiotensin converting enzyme inhibitors; ARB, angiotensin receptor blocker; HbA1c, hemoglobinA1c; CABG, coronary artery bypass grafting.

### 3.2 Association between Different Lipid Parameters and MACEs

**Fig. 1.**
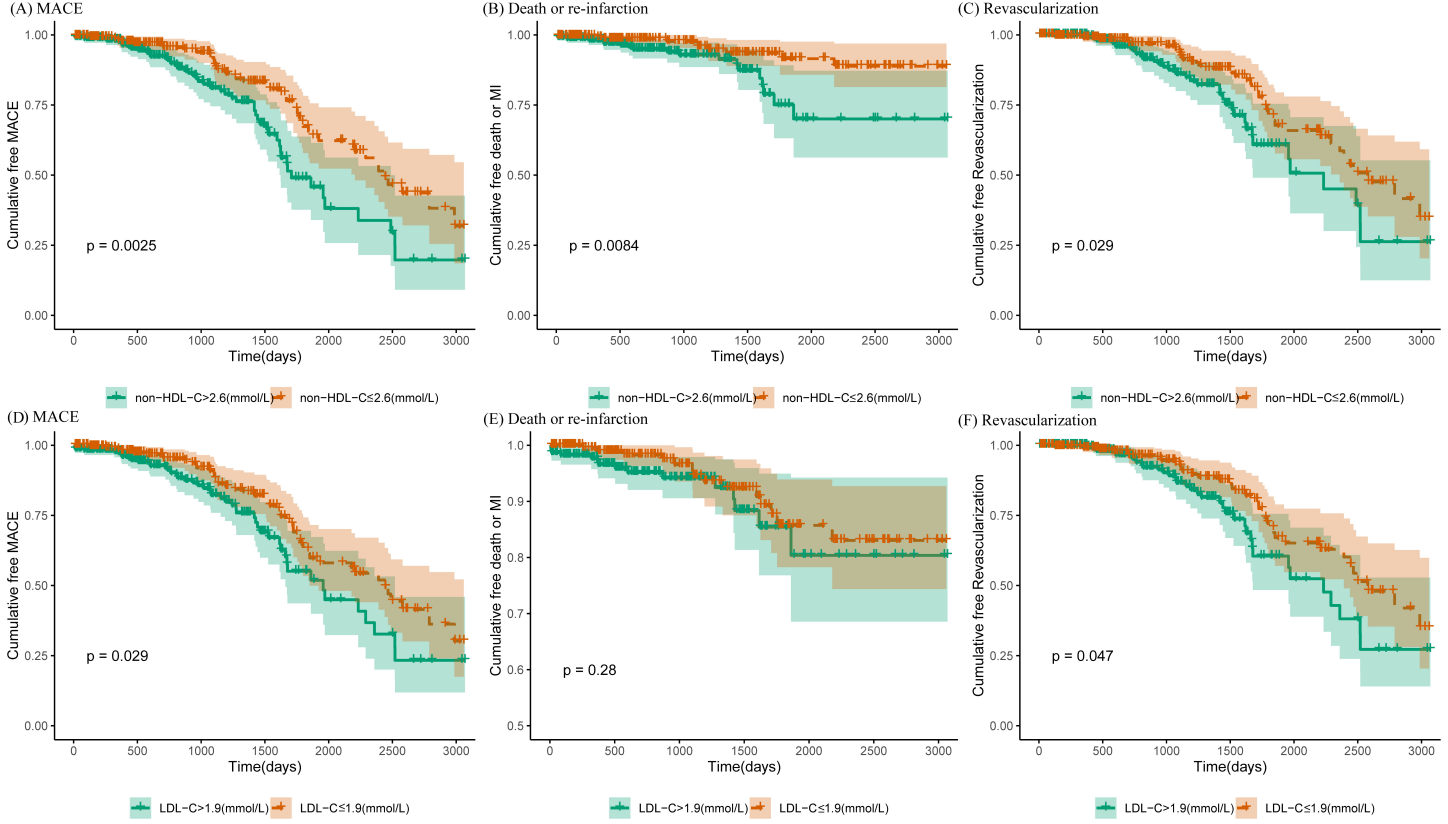
**Kaplan-Meier survival curves for MACE in ACS and statin-treated patients with a history of CABG**. (A–C) Kaplan-Meier survival curves for the incidence of MACE, CV mortality/re-infarction, and revascularization, respectively, in two groups stratified according to the median non-HDL-C level (2.6 mmol/L). (D–F) Kaplan-Meier survival curves for MACE, CV mortality/re-infarction, and revascularization, respectively, between two groups stratified according to the mean LDL-C level (1.9 mmol/L). MACE, major adverse cardiovascular event; CABG, coronary artery bypass grafting; CV, cardiovascular; non-HDL-C, non-high-density lipoprotein cholesterol; LDL-C, low-density lipoprotein cholesterol; ACS, acute coronary syndrome.

Over the median follow-up period of 744.5 days, 95 (20.2%) patients suffered MACEs, comprising 15 (3.2%) CV deaths, 16 (3.4%) recurrent non-fatal Mis, and 72 (15.4%) repeated revascularizations. The survival curves for MACE, CV death/re-infarction, and repeated revascularization in patients with high non-HDL-C levels declined lowlier than the ones with low non-HDL-C (Log-rank test: *p* = 0.0025, 0.0084, and 0.029, respectively, shown in Fig. 1). When the median LDL-C value stratified the cohort, similar trends were observed in high LDL levels regarding MACE and revascularization (Log-rank test: *p* = 0.029 and 0.047, respectively) whereas none for the incidence of CV death/re-infarction.

Table 3 shows the association between the lipid parameters and MACE performing Cox regression analyses. In Model 1, after adjustment for confounding factors (age, gender, BMI, left ventricular ejection fraction (LVEF), peak level of cardiac troponin I (cTnI)), multivariate analysis indicated that non-HDL-C exhibits a superior to LDL-C for the prediction of MACEs (HRs = 1.62 per 1-SD increment in non-HDL-C level *vs.* 1.50 per 1-SD increment in LDL-C level), death/re-infarction (HRs = 1.88 per 1-SD increment in non-HDL-C level *vs.* 1.82 per 1-SD increment in LDL-C level), and revascularization (HRs = 1.48 per 1-SD increment in non-HDL-C level *vs.* 1.37 per 1-SD increment in LDL-C level). Similar associations were observed when vein grafting PCI, clinical vital signs, and creatinine clearance rates were incorporated into Model 2, which controlled for the confounders identified in Model 1. Additionally, in Model 3, the predictive ability of these lipid indices was analyzed after adjustment for statin treatment and use of beta-blockers as confounding factors in addition to Model 2. Even though those parameters related to CV outcomes in patients with previous CABG were taken into consideration, the non-HDL-C level retained its advantageous predictive probability regarding the occurrence of MACE, CV deaths/re-infarction, and revascularization compared with that of the other lipid parameters (HRs = 1.52, 1.65, and 1.42 per 1-SD increment in non-HDL-C levels for the three outcomes, respectively). Regarding the multicollinearity, the VIF lower than 10 in Model 3 indicated the absence of strong interactions between the lipid variables and other confounding factors.

**Table 3.** ** Comparisons among different lipid indices estimated by multivariate Cox proportional-hazard regression regarding major adverse cardiovascular events**.

Variables		Model $1^{\#}$		Model 2		Model 3*	
		HRs (95% CIs)	*p*-value	HRs (95% CIs)	*p*-value	HRs (95% CIs)	*p*-value
MACE							
	Non-HDL-C	1.62 (1.31, 2.00)	<0.001	1.52 (1.24, 1.88)	<0.001	1.52 (1.24, 1.87)	<0.001
	LDL-C	1.50 (1.21, 1.87)	<0.001	1.40 (1.13, 1.74)	<0.001	1.44 (1.14, 1.73)	<0.01
	TC	1.49 (1.20, 1.86)	<0.001	1.44 (1.16, 1.78)	<0.001	1.44 (1.17, 1.78)	<0.001
	TG	1.32 (1.10, 1.61)	<0.01	1.34 (1.10, 1.64)	<0.01	1.36 (1.11, 1.67)	<0.01
	HDL-C	0.77 (0.61, 0.96)	<0.05	0.82 (0.66, 1.03)	0.08	0.83 (0.66, 1.04)	0.11
	Lp(a)	1.10 (0.82, 1.46)	0.52	1.09 (0.81, 1.48)	0.55	1.10 (0.82, 1.48)	0.51
Death or re-infarction							
	Non-HDL-C	1.88 (1.34, 2.64)	<0.001	1.76 (1.26, 2.43)	<0.001	1.65 (1.17, 2.33)	<0.01
	LDL-C	1.82 (1.27, 2.61)	<0.01	1.67 (1.18, 2.35)	<0.01	1.57 (1.10, 2.24)	<0.05
	TC	1.86 (1.31, 2.63)	<0.001	1.74 (1.25, 2.42)	<0.001	1.65 (1.16, 2.34)	<0.01
	TG	1.26 (0.87, 1.83)	0.22	1.28 (0.85, 1.94)	0.23	1.27 (0.82, 1.97)	0.29
	HDL-C	0.98 (0.68, 1.41)	0.94	0.97 (0.69, 1.38)	0.88	0.99 (0.68, 1.43)	0.96
	Lp(a)	1.18 (0.80, 1.74)	0.40	1.23 (0.89, 1.68)	0.20	1.22 (0.88, 1.71)	0.22
Revascularization							
	Non-HDL-C	1.48 (1.14, 1.91)	<0.01	1.42 (1.10, 1.82)	<0.01	1.42 (1.12, 1.83)	<0.01
	LDL-C	1.37 (1.05, 1.78)	<0.001	1.31 (1.01, 1.70)	<0.05	1.32 (1.03, 1.70)	<0.05
	TC	1.30 (0.99, 1.71)	0.06	1.29 (1.00, 1.68)	0.05	1.32 (1.02, 1.71)	<0.05
	TG	1.32 (1.06, 1.64)	<0.05	1.32 (1.05, 1.67)	<0.05	1.34 (1.06, 1.70)	<0.05
	HDL-C	0.68 (0.53, 0.90)	<0.01	0.74 (0.56, 0.97)	<0.05	0.75 (0.57, 0.98)	<0.05
	Lp(a)	0.99 (0.68, 1.44)	0.92	0.94 (0.63, 1.42)	0.79	0.96 (0.64, 1.44)	0.86

#, Model 1, adjusted for conventional coronary risk variables including age, sex, BMI, LVEF, peak level of cTnI; Model 2, adjusted for clinical risk factors including age, sex, BMI, LVEF, peak level of cTnI, values of heart rates, SBP and DBP at discharge, vein grafting PCI and levels of CCR; Model 3, adjusting for use of statin and β-blocker in addition to Model 2 at baselines. HRs, hazard ratios were calculated per 1-SD for increment in each lipid/apoprotein. MACE, major adverse cardiovascular event; HDL-C, high-density lipoprotein cholesterol; LDL-C, low-density lipoprotein cholesterol; TC, total cholesterol; TG, triglyceride; Lp(a), lipoprotein a; BMI, body mass index; LVEF, left ventricular ejection fraction; cTnI, cardiac troponin I; SBP, systolic blood pressure; DBP, diastolic blood pressure; PCI, percutaneous coronary intervention; CCR, creatinine clearance rate; HR, hazard ratio. *, VIF <10, VIF, variance inflation factor.

### 3.3 RCS and Discordance Analysis

**Fig. 2.**
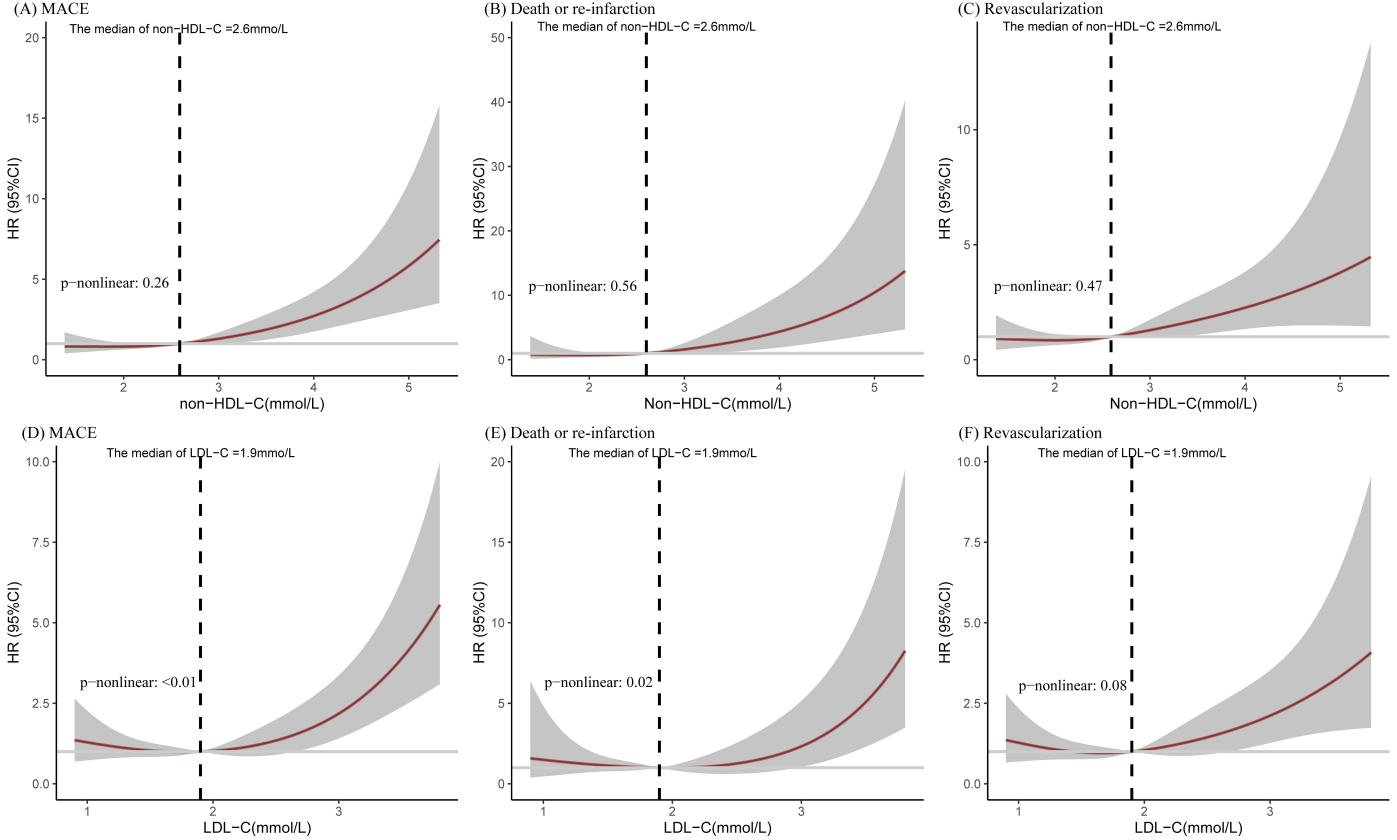
**The restricted cubic spline regression lines between two lipid parameters and CV events**. (A–C) A roughly linear relationship was evidenced for non-HDL-C levels and MACEs, CV death/re-infarction, and revascularization, respectively. (D–F) A roughly nonlinear relationship was presented for LDL-C levels and MACE, CV death/re-infarction, and revascularization, respectively. CV, cardiovascular; non-HDL-C, non-high-density lipoprotein cholesterol; MACEs, major adverse cardiovascular events; LDL-C, low-density lipoprotein cholesterol; HR, hazard ratio.

**Fig. 3.**
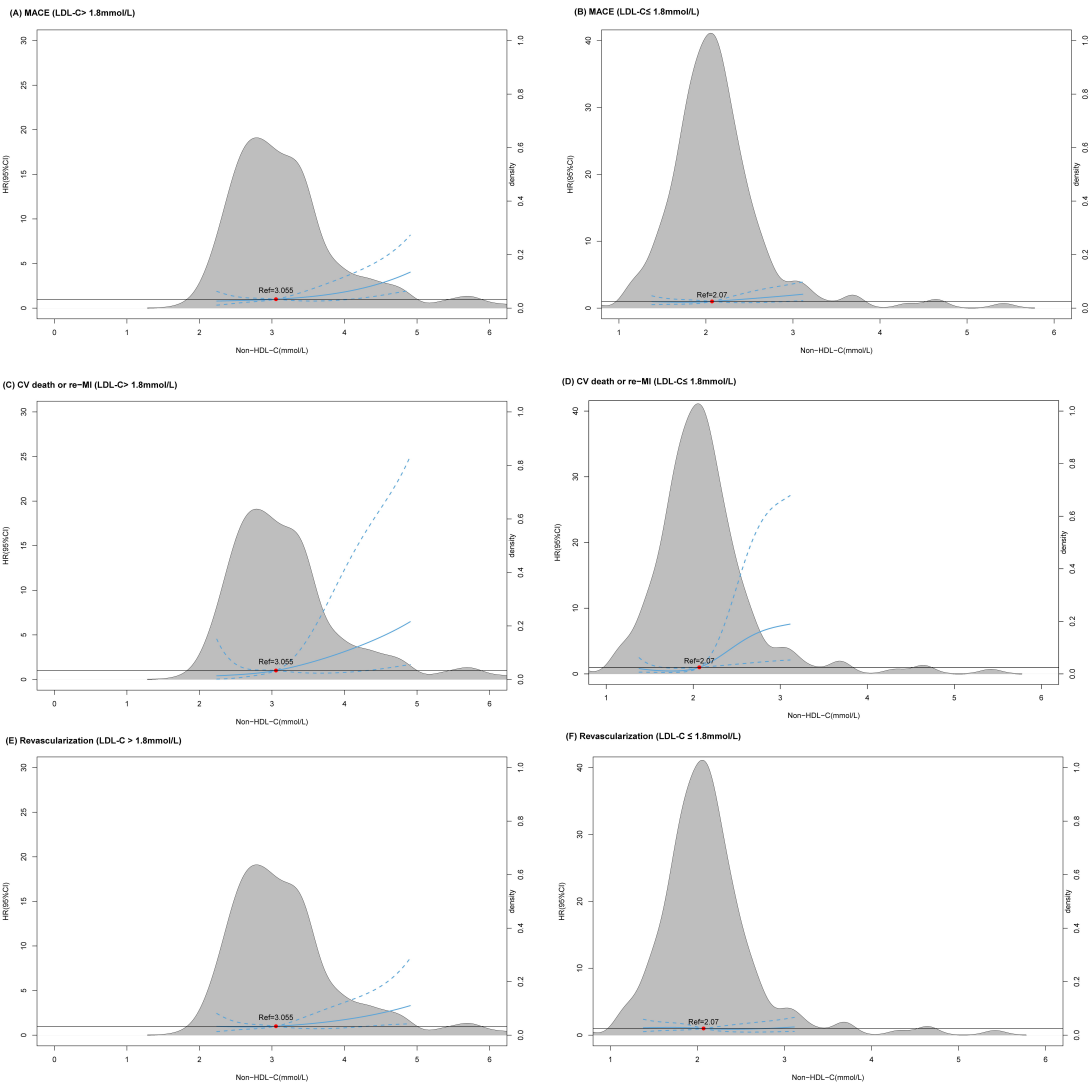
**The restricted cubic spline lines between non-HDL-C levels and the occurrence of MACEs, CV death/re-infarction, and revascularization stratified according to LDL-C levels**. (A, C, E) In patients with high LDL-C levels(>1.8mmol/L), a marginally linear relationship express between non-HDL-C and MACEs, CV death/re-MI and revascularization. (D) In patients with low LDL-C levels (≤1.8 mmol/L), a linear relationship is observed between elevated non-HDL-C levels (>2.07 mmol/L) and the risk of CV death/recurrent MI. (B, F) However, none linear linkage presents between non-HDL-C and MACEs and revascularization. Non-HDL-C, non-high-density lipoprotein cholesterol; MACEs, major adverse cardiovascular events; LDL-C, low-density lipoprotein; MI, myocardial infarction; HR, hazard ratio; CV, cardiovascular.

**Fig. 4.**
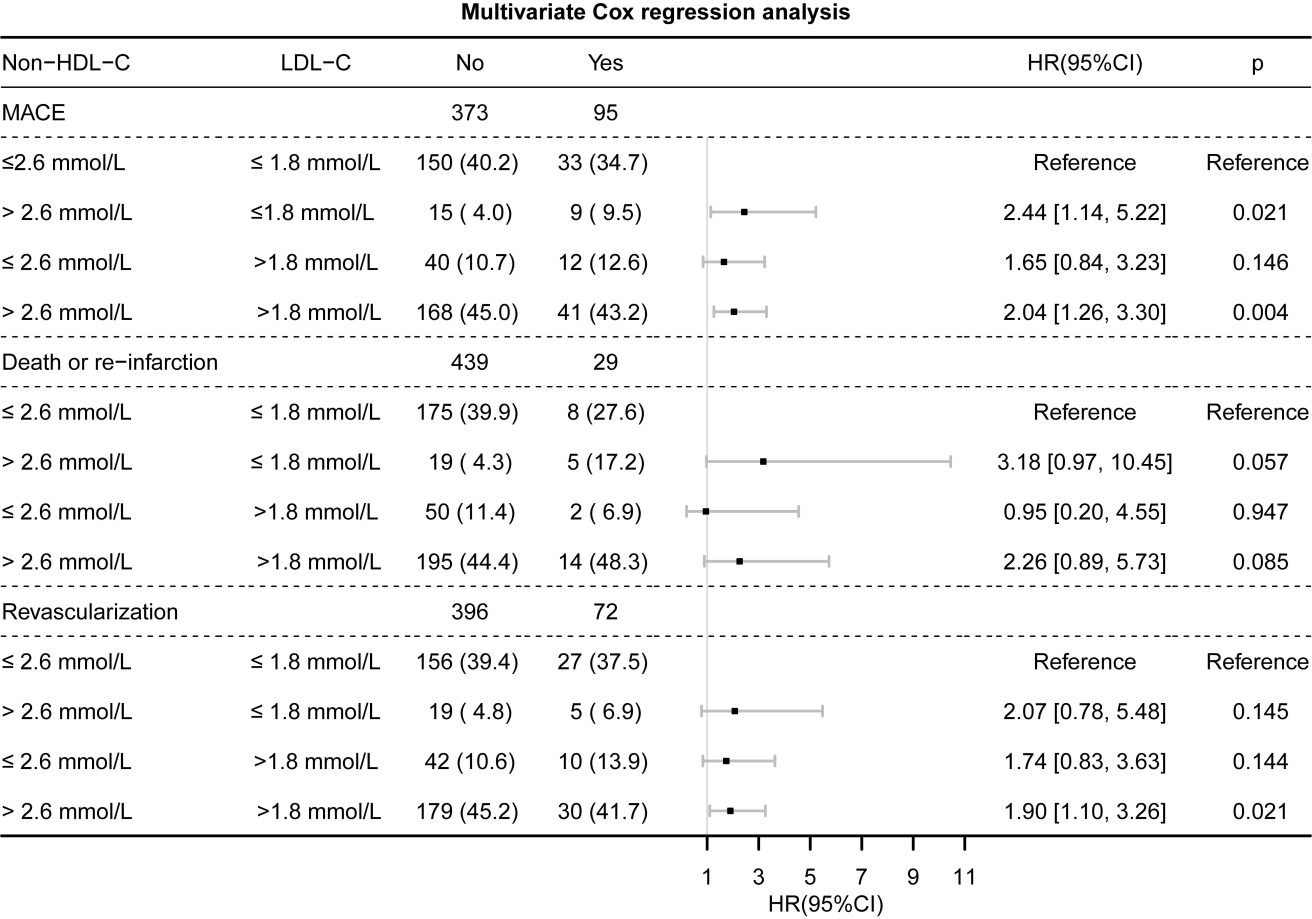
**Multivariate Cox regression analysis for the occurrence of MACEs, CV death/re-infarction, and revascularization in the discordant versus concordant groups based on LDL-C and non-HDL-C levels**. The analysis is adjusted for age, sex, body mass index (BMI), left ventricular ejection fraction (LVEF), peak level of cardiac troponin I (cTnI), heart rate, systolic blood pressure (SBP), and diastolic blood pressure (DBP) at discharge, and creatinine clearance rate (CCR). MACEs, major adverse cardiovascular events; CV, cardiovascular; LDL-C, low-density lipoprotein cholesterol; non-HDL-C, non-high-density lipoprotein cholesterol; HR, hazard ratio.

RCS was utilized to smoothly explore and visualize the relationships between non-HDL-C/LDL-C levels and MACE (Fig. 2). A linear curve was detected between non-HDL-C levels and the risk of MACEs in post-CABG patients with statin and secondary PCI treatment, whereas a nonlinear relationship was observed for LDL-C levels. When circulating non-HDL-C levels exceeded 2.6 mmol/L, a marginally linear risk of suffering MACE, CV death/re-infarction, and revascularization was observed. In addition, RCS was used to investigate the relationship between serum levels of non-HDL-C and MACEs at two different LDL-C levels (Fig. 3). In those individuals with LDL-C levels ≤1.8 mmol/L, a linear association with CV death/re-infarction appeared when non-HDL-C reached up to 2.07 mmol/L. Finally, the discordance analysis indicated that individuals with discordantly high non-HDL-C/low LDL-C had HRs of 2.44 [95% confidence interval (CI), 1.14–5.22, *p* = 0.021], 3.18 (95% CI, 0.97–10.45, *p* = 0.057) and 2.07 (95% CI, 0.78–5.48, *p* = 0.145) for MACEs, CV death/re-infarction and revascularization, respectively, compared to those in patients with concordant non-HDL-C and LDL-C levels (Fig. 4).

## 4. Discussion

The primary findings of this retrospective study shed some valuable insight on the predictive probability of non-HDL-C levels for evaluating the residual risk of long-term outcomes in ACS individuals with a history of CABG and statin therapy. The measurement of non-HDL-C levels better reflects the residual risk of CV events in this population than LDL-C levels. Interestingly, a discordance in lipid levels involving elevated non-HDL-C and low LDLC levels, but not one involving low non-HDL-C and high LDL-C levels, was significantly in association with an elevated likelihood of MACEs among ACS patients with previous CABG and statin treatment that underwent secondary coronary interventions.

A large body of previous studies has assessed the ability of non-HDL-C regarding the prediction of cardiac mortality and other events for the prevention of CV disease [3, 19, 20]. The increased concentration of small dense LDL particles, a reduction in HDL-C levels, the presence of hypertriglyceridemia, an increase in remnant lipoproteins, and postprandial hyperlipidemia were shown to be factors that partially accounted for the mechanisms underlying the residual CV risk observed in some individuals with optimal LDL-C levels [21]. These findings revealed that an increase in non-HDL-C levels indicated the residual risk of MACEs in statin-treated, ACS individuals and previous CABG. Comparable findings were reported by Fukushima *et al*. [15], which indicated that individuals with increased non-HDL-C levels following CABG struggled with significantly higher odds of CV death and other adverse clinical outcomes. However, that study failed to consider the risks associated with previous statin therapy. Similar findings from other studies have manifested that the possibility of serum non-HDL-C for the prediction of clinical prognosis was steadier and more enduring than other lipid parameters [8, 10]. On the other hand, Nakamura Y *et al*. [22] indicated that optimal medical treatments combined with statin treatment, antiplatelet agent, and beta-blockers reduced all-cause death and cardiac death in patients with a history of CABG who underwent PCI. However, in this study, we report a linear curve between the incidence of long-term cardiac events and non-HDL-C concentrations in the setting of moderate-intensity statin therapy for post-CABG patients that required PCI treatment. More specifically, non-HDL-C concentrations exceeding 2.6 mmol/L were likely to be powerful and accurate risk indications of CV prognosis, similar to the results reported by Brunner *et al*. [19]. According to our results from the subgroup analysis, the threshold concentration of non-HDL-C as a risk modifier in statin-treated individuals with optimal LDL-C values would be closer to 2.07 mmol/L. For the participants with undesirable LDL-C levels, only a relatively moderate association was observed between non-HDL-C levels and the risks of CV mortality, recurrence of MI, and the need for revascularization. Thus, achieving target LDL-C concentrations in those patients remains the primary goal for reducing risk in the initial management of patients with previous CABG. These findings show that evaluating non-HDL-C could represent a secondary means of appraising residual risk regarding CV events and identifying extremely high-risk patients with a history of CABG who may require intensification of lipid-lowering therapies.

Patients with a history of CABG typically exhibit a complicated and diffuse progression of atherosclerosis and other complications (e.g., ischemia stroke, diffuse peripheral arterial atherosclerosis, serious renal insufficiency, and rheumatology disease) and struggle with more fatal and non-fatal events [23, 24, 25]. These findings indicated in this population that discordance between low LDL-C and high non-HDL-C levels was observed in up to 5.1% of cases, similar to the proportions reported by the previous studies [26, 27]. In a recent Johannesen *et al*. [10] study, a discordance involving low LDL-C/ high non-HDL-C or apo(B) was associated with a 91% higher risk of MI and a 23% higher risk of mortality compared with the risk in those with concordantly low levels of both factors; a similar trend was not observed in those exhibiting a discordance involving high LDL-C and low non-HDL-C levels. Several explanations have been provided for this phenomenon, which is prevalently consistent with the previous studies [8, 10]. Firstly, non-HDL cholesterol fraction encompasses circulating LDL-C, very low-density lipoprotein (VLDL) cholesterol, and other ingredients from byproducts of triglyceride-related lipoprotein metabolism. Increased levels of atherogenic cholesterol are superior indicators of the mass of lipoprotein particles in evaluating associations that affect ASCVD risk [3]. This discordance may reflect higher concentrations of remnant cholesterol, which could lead to an atherosclerotic cardiovascular disease beyond apolipoprotein B and the significant amounts of atherogenic LDL particulates that interact with the coronary artery. It is not enough to measure the concentrations of these circulating lipoprotein components [5, 28]. In addition, the discordance between two cholesterol parameters was mediated by metabolic syndrome and unrelated to the conventional risk factors regardless of the BMI and the degree of glycemic control [5, 10], as the findings of Model 3 were indicative of the absence of multicollinearity. In patients exhibiting a discordance involving low LDL-C and high apolipoprotein B or non-HDL-C levels, an increased risk of arterial stiffness was observed, as measured by brachial-ankle pulse wave velocity; these factors are regarded as markers of subclinical atherosclerosis [27, 29]. In general, elevated arterial stiffness and multifocal atherosclerosis in patients after CABG tracked more with the atherosclerosis progression and a poor prognosis [30, 31, 32]. Although the biological mechanisms underlying the causality of these relationships remain inexplicable, those exhibiting a discordance between two lipid parameters presented elevated levels of remnant TGs or cholesterol related to insulin resistance. In turn, this contributed to increased arterial stiffness and the development of diffuse and multifocal atherosclerosis because of the disorder of intimal cells caused by varying degrees of oxidative responses and impaired endothelial function [11, 33]. In this study, the subgroup of patients with a CABG history with elevated non-HDL-C and optimal LDL-C levels exhibited a risk of CV death or re-infarction that was as much as four times higher than that observed in the group with concordant lipid levels confirming that further efforts are needed in such patients to ensure enhanced low-lipid management.

## 5. Limitations

In addition to the inevitable selection bias inherent to retrospective studies, several limitations existed in this study. First, the small example size and relatively low incidence of CV death/re-infarction in the enrolled patients may have contributed to a high selection bias that could complicate the interpretation of the results. A comparable prevalence of CV deaths is reported in another real-world Chinese research [34]. Thus, it is possible that any inconsistencies could be related to the ethnic makeup of the group and could provide some insight into the predictive value of lipid discordance on prognosis in Chinese patients with a history of CABG. Second, apolipoprotein B measurements were not performed in this study because of the limitations of our laboratory; therefore, the analysis was limited to comparing groups based on LDL-C and non-HDL-C levels. Based on the high correlation between these two parameters, the Adult Treatment Panel III (ATP III) guidelines recommend using non-HDL-C levels as a reasonable substitute for apolipoprotein B concentrations [35]. Third, approximately half of the participants in this study did not reach the optimal LDL-C levels recommended by the guidelines, which could have influenced the credibility of the results. Therefore, a subgroup analysis was conducted that was stratified based on the median LDL-C concentration to classify the impact of non-HDL-C levels on prognosis. Large-scale research is necessary to explore the effect of lipid discordance on saphenous grafting and clinical prognosis in post-CABG patients with statin treatment who underwent PCI treatment.

## 6. Conclusions

In post-CABG and statin treated ACS individuals, who received secondary PCI, there is a linear association of non-HDL-C with the significant risk of MACE. Moreover, a linear relationship between non-HDL-C values exceeding 2.07 mmol/L and risks of CV death/recurrent MI was presented in those patients with desirable LDL-C levels (≤1.8 mmol/L). The discordance of high non-HDL-C/low LDL-C could provide utility in identifying the residual risk of MACEs in this population.

## Data Availability

The datasets generated and analyzed are not publicly available due to the policies of Beijing Chaoyang Hospital regarding individual confidentiality; however, they are available from the corresponding author upon reasonable request.

## Abbreviations

MACE, major adverse cardiovascular events; CAD, coronary artery disease; PCI, percutaneous coronary intervention; GRACE, Global Registry of Acute Coronary Events; MI, myocardial infarction; CABG, coronary artery bypass grafting; TC, total cholesterol; LDL-C, low-density lipoprotein cholesterol; HDL-C, high-density lipoprotein cholesterol; TG, triglyceride; Lp(a), apolipoprotein A; CCR, creatinine clearance rate; ESR, erythrocyte sedimentation rate; Hs-CRP, high-sensitivity C-reactive protein; cTnI, cardiac troponin I; CKMB, creatine kinase–myocardial band; BMI, body mass index; LVEF, left ventricular ejection fraction; ROC, receiver operating characteristic; AUC, area under the ROC.

## Supplementary Material

Supplementary material associated with this article can be found, in the online version, at https://doi.org/10.31083/j.rcm2409263.
