# Supplementary figures and images for: Discordance of Circulating Non-HDL Cholesterol with LDL Cholesterol Concerning Long-Term Prognosis in Statin-Treated Individuals with Acute Coronary Syndrome and Previous Coronary Artery Bypass Grafting Undergoing Percutaneous Coronary Intervention
Source: Rev Cardiovasc Med. 2023 Sep 21;24(9):263. doi: 10.31083/j.rcm2409263 (PMC11270103; doi:10.31083/j.rcm2409263)

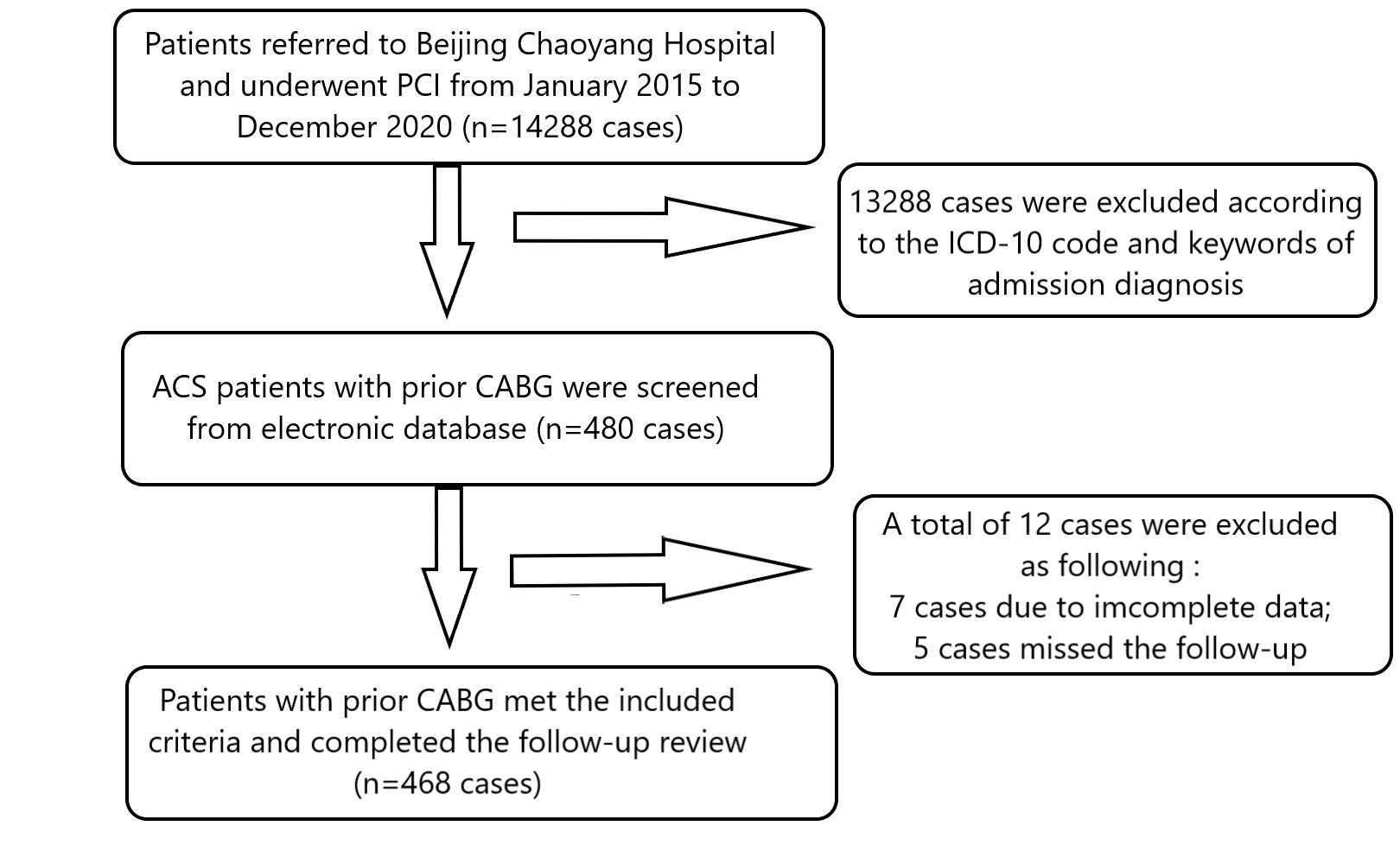

Supplement: Supplementary file 1 [file 2153-8174-24-9-263-s1.zip › 2153-8174-24-9-263-s1.png]
